# Synergic Effect of Allopurinol in Combination with Nitroheterocyclic Compounds against Trypanosoma cruzi

**DOI:** 10.1128/AAC.02264-18

**Published:** 2019-05-23

**Authors:** Ana Lia Mazzeti, Lívia de F. Diniz, Karolina R. Gonçalves, Ruan Schott WonDollinger, Tassiane Assíria, Isabela Ribeiro, Maria T. Bahia

**Affiliations:** a Laboratório de Doenças Parasitárias, Escola de Medicina & Núcleo de Pesquisas em Ciências Biológicas, Universidade Federal de Ouro Preto, Campus Universitário Morro do Cruzeiro, Ouro Preto, Minas Gerais, Brazil; b Laboratório de Parasitologia Básica, Departamento de Patologia e Parasitologia, Instituto de Ciências Biomédicas, Universidade Federal de Alfenas, Alfenas, Minas Gerais, Brazil; c Laboratório de Imunopatologia, Fundação Oswaldo Cruz, Centro de Pesquisa René Rachou, Belo Horizonte, Minas Gerais, Brazil; d Drugs for Neglected Diseases Initiative, Geneva, Switzerland

**Keywords:** Chagas disease, allopurinol, benznidazole, chemotherapy, combination therapy, nitro compounds

## Abstract

Combination therapy has gained attention as a possible strategy for overcoming the limitations of the present therapeutic arsenal for Chagas disease. The aim of this study was to evaluate the effect of allopurinol in association with nitroheterocyclic compounds on infection with the Y strain of Trypanosoma cruzi. The *in vitro* effect of allopurinol plus benznidazole or nifurtimox on intracellular amastigotes in infected H9c2 cells was assessed in a 72-h assay. The interactions were classified as synergic for both allopurinol-nifurtimox (sums of fractional inhibitory concentrations [∑FICs] = 0.49 ± 0.08) and allopurinol-benznidazole (∑FICs = 0.48 ± 0.09). In the next step, infected Swiss mice were treated with allopurinol at 30, 60, and 90 mg/kg of body weight and with benznidazole at 25, 50, and 75 mg/kg in monotherapy and in combination at the same doses; as a reference treatment, another group of animals received benznidazole at 100 mg/kg. Allopurinol in monotherapy led to a smaller or nil effect in the reduction of parasite load and mortality rate. Treatment with benznidazole at suboptimal doses induced a transient suppression of parasitaemia with subsequent relapse in all animals treated with 25 and 50 mg/kg and in 80% of those that received 75 mg/kg. Administration of the drugs in combination significantly increased the cure rate to 60 to 100% among mice treated with benznidazole at 75 mg/kg plus 30, 60, or 90 mg/kg of allopurinol. These results show a positive interaction between allopurinol and benznidazole, and since both drugs are commercially available, their use in combination may be considered for the assessment in the treatment of Chagas disease patients.

## Abstract (precis)

Combination therapy has gained attention as a possible strategy for overcoming the limitations of the present therapeutic arsenal for Chagas disease. The aim of this study was to evaluate the effect of allopurinol in association with nitroheterocyclic compounds on infection with the Y strain of Trypanosoma cruzi.

## INTRODUCTION

American trypanosomiasis, or Chagas disease, is a neglected disease caused by the protozoan Trypanosoma cruzi. Currently about 7 million people are infected worldwide, especially in Latin America (1). The etiological treatment available for Chagas disease is based on nitroheterocyclic compounds (benznidazole and nifurtimox) and is effective in treating acute and early chronic phases, but it has less established efficacy when administered during the chronic phase of the disease (2). Moreover, both compounds function as prodrugs, and their administration can be associated with safety and tolerability issues (3).

Drug repositioning could be a source of alternative chemotherapies for Chagas disease. This strategy of finding new uses for existing drugs is beginning to yield results: anti-T. cruzi activity has been discovered for existing drugs used for cancer (4, 5), fungal diseases (6–15), and hyperuricemia (16–18). Profile-based repositioning strategies have successfully identified allopurinol, an alternative substrate for parasite hypoxanthine-guanine phosphoribosyltransferase, as a possible new drug candidate for Chagas disease (17, 19). A number of studies have demonstrated the anti-T. cruzi activity of allopurinol: *in vitro* (20, 21), in murine models of acute infection (16, 18), and in the treatment of individuals in the chronic phase of Chagas disease from Chile and Argentina (22–24). Others have demonstrated the efficacy of allopurinol in treating Chagas disease reactivation after heart transplantation (25). Despite these results, allopurinol is not efficacious in the control of parasitemia of acute Chagas disease patients in monotherapy (26) or in curing patients in the chronic phase of Chagas disease from areas of endemicity in Brazil (27). These findings highlight the need to investigate alternative dosing regimens and possible combination therapies to improve the efficacy of allopurinol in treating Chagas disease. Grosso et al. (28) evaluated the effect of sequential treatment with allopurinol and benznidazole in a murine experimental model of acute and chronic T. cruzi infection. This study showed that the administration of allopurinol immediately after benznidazole treatment was able to reduce parasitemia and attenuate tissue damage by reducing heart muscle inflammation. Others have also demonstrated the benefits of sequential combined administration of allopurinol and benznidazole in human chronic disease, which leads to changes in T and B immune responses indicative of beneficial therapeutic outcomes (29). Similarly, an improvement in the treatment of cutaneous leishmaniasis also has been observed when allopurinol was given in combination with antimony compounds (30, 31) or trichloroacetic acid (32). Rial et al. (33) evaluated the effect of the allopurinol combined with benznidazole in C3H/HeN and C57BL/6J mice with T. cruzi infection with the Nicaragua strain and Sylvio-X10/4 clone. Animals were treated during the chronic phase (3 months of infection) with 50 or 100 mg/kg of body weight of benznidazole administered for 30 consecutive days or intermittently with one treatment dose every 7 days (13 doses), associated or not with 64 mg/kg of allopurinol. In that study, allopurinol addition to the lowest dose of benznidazole had a positive interaction on serology and pathology in TcN-C57BL/6J mice and on pathology in TcSylvio-X10/4-C3H/HeN mice. However, in this study, the effect of treatment with allopurinol alone was not shown, which makes it difficult to interpret the effect of this combination of drugs.

Given this context, the objective of this study was to evaluate the effect of using allopurinol in combination with nitro compounds on *in vitro* and *in vivo* infection with T. cruzi.

## RESULTS

### Allopurinol-nitroheterocyclic drug *in vitro* interaction.

**FIG 1:**
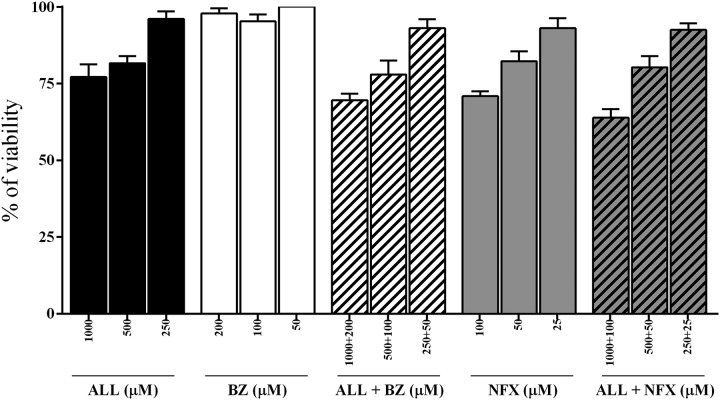
*In vitro* viability after treatment with allopurinol (ALL), benznidazole (BZ), and nifurtimox (NFX) alone or in combination. Shown is percent viability of H9c2 cells incubated for 72 h at different concentrations of allopurinol (ALL), benznidazole (BZ), and nifurtimox (NFX) and with ALL-BZ and ALL-NFX combinations.

In order to identify possible cytotoxicity of the allopurinol-nitroheterocyclic combination therapy, the viability of uninfected host cells in the presence of compounds alone and in combination was evaluated *in vitro* using the H9c2 cell line. Figure 1 shows cell viability 72 h after different treatments. Our data showed that allopurinol, benznidazole, and nifurtimox preserve cell viability, even at the highest concentrations. On the other hand, combinations of allopurinol-benznidazole and allopurinol-nifurtimox demonstrated an additional toxic effect on H9c2 cell proliferation at the highest concentrations of the combined drugs (1,000 + 200 μM and 1,000 + 100 μM, respectively). These concentrations were not used in the anti-T. cruzi activity assays.

**FIG 2:**
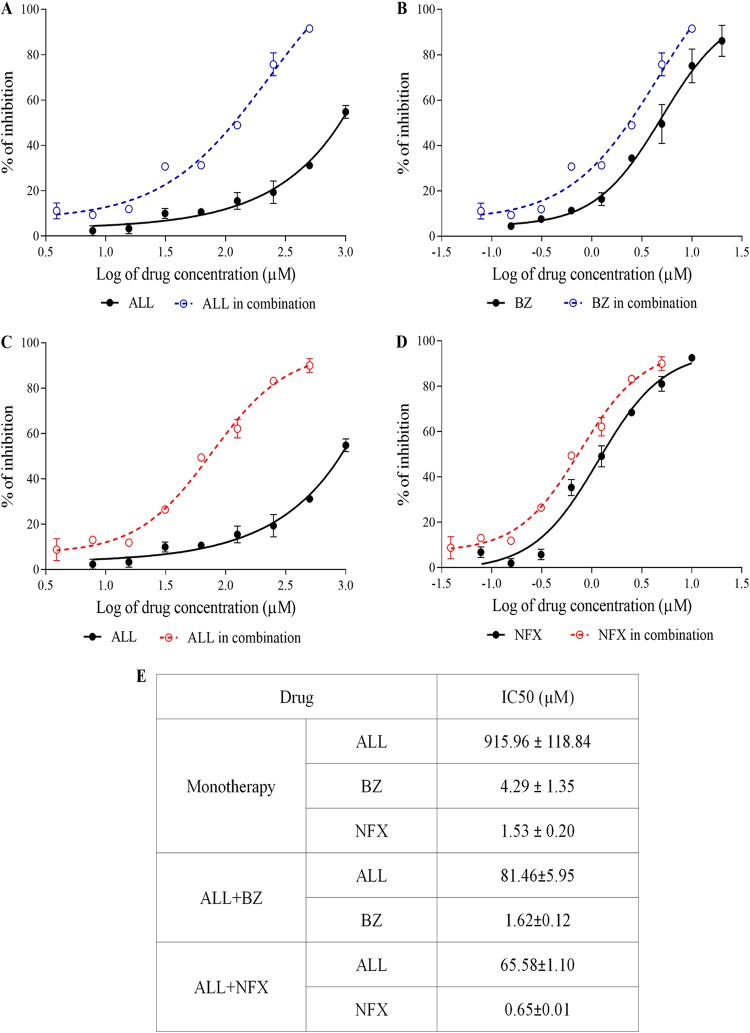
Dose-response curves for ALL and BZ or NFX against amastigote forms of the Y strain of Trypanosoma cruzi, using cardiomyoblast derivative H9c2 as host cells. (A) ALL alone and combined with BZ. (B) BZ alone and combined with ALL. (C) ALL alone and combined with NFX. (D) NFX alone and combined with ALL. (E) The drug concentration alone or in combination required to kill 50% of the parasite population (IC_50_).

**FIG 3:**
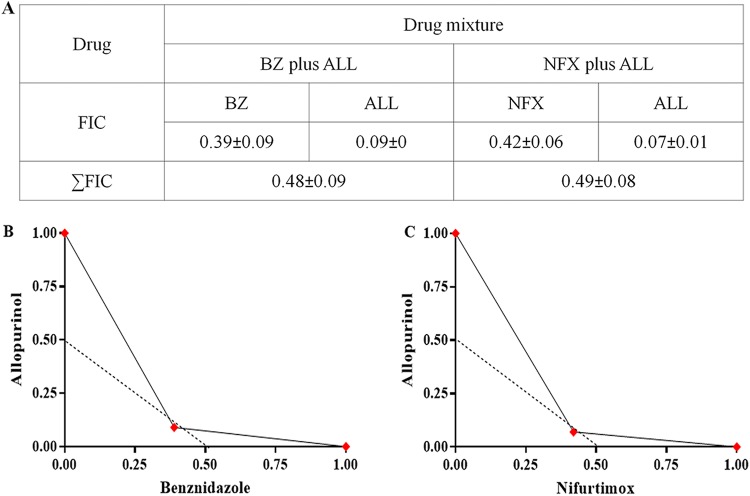
*In vitro* interaction of allopurinol with nitroheterocyclic compounds. (A) Fifty percent fractional inhibitory concentration (FIC_50_) of each drug in combination and ∑FIC_50_ of allopurinol plus benznidazole and allopurinol plus nifurtimox. (B) Graphical representations of the interaction between allopurinol and benznidazole. (C) Graphical representations of the interaction between allopurinol and nifurtimox.

The nature of the interaction of allopurinol and nitroheterocyclic drugs on T. cruzi *in vitro* was assessed. Initially, the activity of the drugs alone on intracellular parasites using H9c2 host cells was determined. The drugs showed a concentration-dependent reduction in the number of infected cells with all compounds evaluated (Fig. 2A to D). The 50% inhibitory concentrations (IC_50_s) detected for benznidazole (4.29 ± 1.35 μM) and nifurtimox (1.53 ± 0.20 μM) were significantly lower than that for allopurinol (915.96 ± 84.04 μM), showing the high potency of these nitro compounds (Fig. 2E). Next, the *in vitro* allopurinol-benznidazole and allopurinol-nifurtimox interactions were assessed using a 1:1 concentration of each drug in a 2-fold serial dilution. The dose-response curves produced by the drug combination suggest an improved trypanocidal effect (Fig. 2A to D), confirmed by a reduction of the IC_50_ compared to each drug alone (Fig. 2E). From the IC_50_, the 50% fractional inhibitory concentration (FIC_50_) of each drug in combination was calculated. Figure 3A presents the mean sums of FICs (∑FICs) of two independent experiments. The interaction of allopurinol with benznidazole and allopurinol with nifurtimox can be classified as synergistic based on the mean ∑FICs of 0.48 ± 0.09 and 0.49 ± 0.08, respectively. The isobols in Fig. 3B and C clearly depict synergistic interactions between the compounds evaluated.

### Allopurinol-benznidazole: *in vivo* interaction.

The therapeutic effect of allopurinol-benznidazole combinations was explored in an established infection with the Y T. cruzi strain, in which all untreated mice presented high levels of parasitemia and mortality occurring on average at 15 to 21 days postinfection (Table 1). Treatment with allopurinol, regardless of dose regimen, led to a smaller or nil effect on parasitemia suppression, reduction of the parasite load (data not shown), and reduction in mortality rate (Table 1). In contrast, benznidazole treatment with suboptimal doses (25, 50, and 75 mg/kg) led to a transient suppression of parasitemia with a subsequent relapse among all animals treated with 25 and 50 mg/kg and in 80% of those that received 75 mg/kg. Even though cure was detected in only 20% of animals treated with 75 mg/kg, benznidazole prevented mortality in Y strain-infected mice, independent of the dose (Table 1). The reference treatment, the optimal dose of 100 mg/kg of benznidazole, induced a 70% cure rate when administered alone for 20 days.

**TABLE 1:** Efficacy of allopurinol in monotherapy or combined with benznidazole in a murine model of acute Trypanosoma cruzi infection^*a*^

Group	Parasitemia clearance (no. of days of treatment)	+FBE^*b*^	+PCR^*c*^	No. of negative results/no. of animals (%)	No. of surviving animals/total (%)^*d*^
NIC			0/5	5/5 (100)	5/5 (100)
IC			ND	0/10 (0)	0/10 (0)
ALL (mg/kg)					
30	0/10	10/10	ND	0/10 (0)	1/10 (10)
60	0/10	10/10	ND	0/10 (0)	0/10 (0)
90	0/10	10/10	ND	0/10 (0)	1/10 (10)
BZ (mg/kg)					
25	7/10 (8.43 ± 3.78)	10/10	ND	0/10 (0)	9/10 (90)
50	10/10 (4.10 ± 3.69)	10/10	ND	0/10 (0)	10/10 (100)
75	8/8 (1.44 ± 0.73)	8/10	0/2	2/10 (20)	10/10 (100)
BZ + ALL (mg/kg)					
25 + 30	8/10 (13.00 ± 5.12)	10/10	ND	0/10 (0)	8/10 (80)
25 + 60	10/10 (10.30 ± 3.74)	8/10	2/2	0/10 (0)	9/10 (90)
25 + 90	10/10 (6.20 ± 4.20)	8/10	2/2	0/10 (0)	10/10 (100)
50 + 30	10/10 (2.50 ± 0.71)	9/10	1/1	0/10 (0)	10/10 (100)
50 + 60	10/10 (2.00 ± 0.67)	8/10	0/2	2/10 (20)	10/10 (100)
50 + 90	9/9 (4.30 ± 3.7)	9/10	1/1	0/9 (0)	9/10 (90)
75 + 30	10/10 (1.66 ± 0.71)	2/10	2/8	6/10 (60)	10/10 (100)
75 + 60	10/10 (2.10 ± 0.32)	0/10	0/10	10/10 (100)	10/10 (100)
75 + 90	10/10 (2.00 ± 0.67)	0/10	0/10	10/10 (100)	10/10 (100)

a Female Swiss (18 to 22 g) were inoculated with 5 × 10^3^ trypomastigotes of T. cruzi strain Y. Treatments were started on the 4th day after infection, by gavage, for 20 consecutive days. NIC, noninfected control; IC, infected control; ND, not done.

b +FBE, positive fresh blood examination during and after treatment (before and after cyclophosphamide immunosuppression).

c +PCR, positive result for PCR assay at 30 and 180 days after treatment.

d Mortality until 30 days after treatment.

For the combinations, allopurinol-benznidazole associations were unable to induce parasitological cure in mice infected with the Y strain when administered at doses of 25 and 50 mg of benznidazole plus 30, 60, and 90 mg of allopurinol for 20 days, since reactivation of parasitemia was detected in 80% to 100% of mice after the end of treatment (Table 1). Additionally, only 20% of animals treated with 50 mg/kg of benznidazole plus 60 mg/kg of allopurinol had negative results in PCR assays performed with blood samples collected 30 and 180 days after treatment. On the other hand, the reactivation of parasitemia was detected in only 20% of animals receiving 75 mg/kg of benznidazole in combination with 30 mg/kg of allopurinol (Table 1). In addition, no animals receiving 75 mg/kg of benznidazole in combination with 60 and 90 mg/kg of allopurinol had positive fresh blood examinations, even after immunosuppression with cyclophosphamide. The results of parasitological and blood PCR assays verified a cure in 60% to 100% of mice receiving 75 mg/kg of benznidazole plus 30, 60, and 90 mg/kg of allopurinol. Such results clearly confirm the synergistic *in vitro* effects of the drug combination, particularly as the effects observed with the combination were three to five times greater than when using benznidazole alone at the same dose. Importantly, the drug combinations were well tolerated, and no mortality was detected among the animals receiving the treatments.

**FIG 4:**
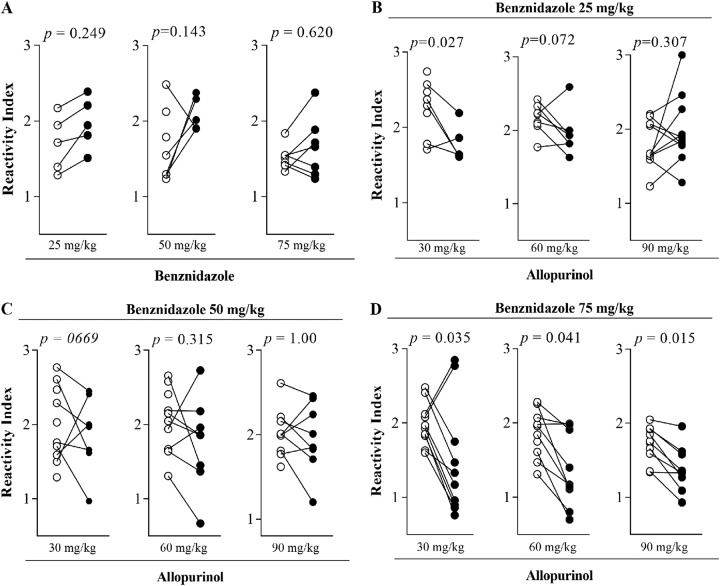
Effects of treatments on IgG antibody levels. Shown are reactivity indices of specific IgG antibodies in serum samples collected from Trypanosoma cruzi-infected mice 30 (open circles) and 180 (closed circles) days after treatment with benznidazole in monotherapy or combined with allopurinol. Note that mice infected and not treated, and those treated with allopurinol in monotherapy, succumbed before the IgG measurement period.

In order to evaluate the effect of specific treatment on the humoral immune response, we measured the levels of IgG anti-T. cruzi antibody in serum samples from the animals. Figure 4 presents the IgG reactivity index determined 30 and 180 days after treatment with allopurinol and benznidazole alone or in combination. The antibody levels remained stable in most sample sera of animals treated with 25, 50, and 75 mg of benznidazole alone and in those that received the combined treatment of 25 and 50 mg of benznidazole combined with all doses of allopurinol (Fig. 4A to C). In contrast, sera obtained from animals treated with 75 mg of benznidazole plus 30, 60, and 90 mg/kg of allopurinol exhibited a significant decrease in the anti-T. cruzi antibody levels (Fig. 4D) in the same period. These results agree with the parasitological evaluation, confirming the marked reduction of parasite load induced by the drug combination.

## DISCUSSION

This study demonstrates the successful combination of a repurposed drug with a known trypanocide in the treatment of experimental T. cruzi infection. Drug combination could increase the success rate of drug repurposing screens (34), since the association of molecules with different mechanisms of action may ameliorate the pharmacokinetic profile, reduce the required drug dose, and increase treatment efficacy.

Allopurinol is a hypoxanthine analogue originally developed for the treatment of hyperuricemia. This drug acts as an alternative substrate of the T. cruzi enzyme hypoxanthine-guanine phosphoribosyltranferase. The enzyme can incorporate allopurinol into parasite RNA, creating a nonfunctional nucleotide that blocks *de novo* synthesis of purines, affects protein synthesis, and induces parasite death (19). In this study, we demonstrated that different doses of allopurinol (30, 60, and 90 mg/kg) were not able to reduce the parasitemia or mortality rate of mice infected by T. cruzi strain Y. In contrast, previous studies with a murine model for Chagas disease have shown that treatment with allopurinol was effective in reducing parasitemia and/or modifying the evolution of acute and chronic infection (16, 18, 35). These discrepancies may be related to methodological differences between the studies, such as the strains used for animal infection, time, and route of drug administration.

There is conflicting evidence on the use of allopurinol monotherapy in clinical studies. Lauria-Pires et al. (26) and Rassi et al. (27) report that the drug was ineffective at inducing a parasitological cure in patients in the acute and chronic phases of Chagas disease. Others have demonstrated that administration of allopurinol in the chronic phase of Chagas disease was effective in reducing the rates of positive xenodiagnosis and reduced or even prevented the appearance of electrocardiogram abnormalities (22, 23). The variability in efficacy of allopurinol treatment in these studies, conducted in different geographic areas, could be at least partially explained by differences between the populations of T. cruzi present in each region and methodologies for evaluation of response. Taken together, these studies show that allopurinol has limited efficacy in the treatment of Chagas disease when used as a single drug, and they highlight the need to investigate alternative dosing regimens and possible combination therapies to improve allopurinol treatment efficacy. This drug repurposing approach may help avoid expensive and time-consuming research on the toxicity and biological availability of new drugs for human consumption.

The *in vitro* assays in this study demonstrated synergistic interactions for allopurinol-benznidazole and allopurinol-nifurtimox combinations; these combinations had no additional toxicities. The IC_50_s detected for allopurinol alone were 915.96 ± 84.04 μM. Other studies detected IC_50_s of >300 μM against epimastigote forms of the Tulahuén and Y strains and 34 μM after treatment of macrophages infected with the Y strain (36). The differences between IC_50_s could be related to the different experimental strategies used, in particular the parasite strain, time of drug incubation, and the host cell used; the influence of the host cell on drug activity *in vitro* has been demonstrated for Leishmania donovani (37). Benznidazole and nifurtimox show high potencies *in vitro*, with IC_50_s in the lower micromolar range. These values are in agreement with those in other studies (38). Interestingly, when the drugs were combined, a drastic reduction of IC_50_s was detected, and analysis of the ∑FIC_50_ (0.48 ± 0.09 for allopurinol-benznidazole and 0.49 ± 0.08 for allopurinol-nifurtimox) confirmed the synergistic interaction between allopurinol and nitroheterocyclic compounds.

The positive interaction observed *in vitro* was confirmed in a murine model of T. cruzi infection; the beneficial effect of the combination, initially verified by the reduction in the number of doses required for the suppression of parasitemia, was comparable to those for the same doses of each drug given in monotherapy (Table 1). The beneficial effect was especially striking when a dose of 75 mg/kg of benznidazole was combined with 60 or 90 mg/kg of allopurinol. When a rigorous evaluation of the curative efficacy of drugs used alone or in combination was performed 30 days posttreatment by immunosuppression of treated animals and monitoring the reactivation of Y strain infections with a blood quantitative PCR (qPCR), it was confirmed that the use of allopurinol/benznidazole in combination prevented death and eradicated the parasitic infection with an efficacy (100% cure) superior to that of the reference treatment (70% cure with 100 mg/kg of benznidazole). As the efficacy of the treatment of mouse infection has been related to the parasite strain and the time of infection (39, 40), studies designed to treat mice infected with parasites belonging to other T. cruzi discrete typing units, as well as chronically infected animals, should be considered.

The levels of the IgG class of antibodies we observed were consistent with results of other assays performed. The levels of specific antibodies decreased significantly in animals receiving 75 mg/kg of benznidazole plus different doses of allopurinol. Overall, a decrease in the levels of specific antibodies is related to greater therapeutic success (41). Given the possible interactions that might result from the use of different compounds in combination, it is clear that a synergistic interaction is beneficial. The synergistic effect we observed between allopurinol and nitro compounds may be associated with the drugs’ mechanism of action. This effect suggests the potentiation of the benznidazole mechanism, allowing the use of 25% lower doses of drug while still guaranteeing cure rates at levels superior to those with treatment at the standard dose. These results support the notion that the use of allopurinol in combination with benznidazole could allow reduction of the dose of benznidazole and, at least theoretically, could reduce the side effects of benznidazole. No studies to date have evaluated the impact of lower doses of benznidazole in humans. Pinazo et al. (42) failed to find a relationship between the benznidazole serum concentrations and adverse reactions. In contrast, studies with children documented lower exposures and suggest that these may be associated with improved safety outcomes (43). In this context, a rigorous evaluation of the tolerability of lower doses of benznidazole should be carried out in randomized clinical studies (one such example is the Drugs for Neglected Diseases Initiative [DND*i*]-sponsored BENDITA study, registered at ClinicalTrials.gov under registration number NCT03378661, presently ongoing).

Likewise, the sequential treatment of individuals in the chronic phase of Chagas disease with allopurinol and benznidazole was well tolerated and induced immunological changes that are associated with a reduction in parasitic load (29). Experimentally, the sequential treatment with allopurinol-benznidazole reduced parasitemia, tissue damage, and the level of anti-T. cruzi antibodies (28). These results are in line with other studies that have discussed the need to evaluate combinations of drugs with different mechanisms of action, such as benznidazole-nifurtimox, benznidazole- or nifurtimox-allopurinol, or triazole antifungal agents to improve the efficacy of Chagas disease treatment (33, 44).

Taken together, these results show a positive interaction between allopurinol and benznidazole *in vivo*; these results are especially promising considering that in some combinations 100% cure was observed in Y strain-infected animals. These results can be correlated with *in vitro* assays, in which the nature of the interaction between the two compounds was classified as synergistic.

## MATERIALS AND METHODS

### Parasite.

The T. cruzi Y strain, DTU II (45), previously characterized as partially resistant to benznidazole (46), was used in this study.

### Study drugs.

Allopurinol [4-hydroxypyrazole(3,4-*d*) pyrimidine, Zyloric; GlaxoSmithKline, Brazil], benznidazole (2-nitroimidazole-*N*-benzyl-2-nitro-1-imidazole-acetamide; LAFEPE, Brazil), and nifurtimox [*N*-(3-methyl-1,1-dioxido-4-thiomorpholinyl)-1-(5-nitro-2-furyl) methanimine; donated by the Drug for Neglected Diseases Initiative] were used in this study.

For *in vitro* studies, stock solutions of allopurinol, benznidazole, and nifurtimox were prepared in dimethyl sulfoxide (DMSO) and stored at −20°C. The stock solutions were further diluted to appropriate working concentrations using fresh culture medium. To avoid toxicity to host cells, the final DMSO concentration never exceeded 0.5% (vol/vol). For *in vivo* assays, all compounds were suspended in solution of 0.5% methylcellulose in distilled water.

### Mammalian cell cultures.

For the toxicity and anti-T. cruzi activity assays, the H9c2 myoblastic cell line (American Type Culture Collection [ATCC]; CRL 1446) was used. The cells were grown in Dulbecco’s modified Eagle’s medium (DMEM) supplemented with 10% fetal bovine serum, 1% l-glutamine (2 μM), 100 IU/ml of penicillin, and 0.1 mg/ml of streptomycin at 37°C in an atmosphere of 5% CO_2_. After counting in a Neubauer chamber, the cells were seeded at a density of 1.0 × 10^4^/well into 24-well culture plates (anti-T. cruzi assays) or 1.0 × 10^3^/well into 96-well plates (toxicity assays).

### Cytotoxicity evaluation.

To test the toxic effects of allopurinol, benznidazole, and nifurtimox on the host cells, uninfected H9c2 cells were incubated for 72 h with increasing concentrations of each drug at 2-fold dilutions, covering ranges of 7.81 to 1,000 μM for allopurinol, 1.56 to 200 μM for benznidazole, and 0.78 to 100 μM for nifurtimox, individually or in combination (ratio, 1:1). The cytotoxicity of the combined compounds was assayed by measuring the viability and proliferation of the cells by resazurin colorimetric assay, as described previously (47). A Biochrom Anthos 2010 spectrophotometer was used to quantify the absorbance at wavelengths of 570 nm (oxidized state) and 600 nm (reduced state) after exposure to rezasurin. Results were reported as percent reduction of viability and inhibition of H9c2 cell proliferation compared to those of control cells in the absence of drug. A reduction in cell viability of more than 30% was considered cytotoxic, as recommended by the International Organization for Standardization (48).

### Allopurinol-nitroheterocyclic drug *in vitro* interaction.

The determination of drug interactions against amastigote forms of T. cruzi was assessed using a 1:1 ratio of each drug in combination. Predetermined 50% effective concentrations (ED_50_s) were used to determine the top concentrations of the individual drugs to ensure that the ED_50_ fell near the midpoint of an eight-point 2-fold dilution series. In brief, 24 h after being seeded on coverslips, the cells were infected with T. cruzi Y strain trypomastigotes at a 20:1 ratio of parasites to host cells. The Y strain induced infection of more than 50% of H9c2 cells. After 24 h of incubation, nonadherent parasites were removed by washing with DMEM and the cultures were exposed to compounds alone or in combination at concentrations ranging from 7.81 to 1,000 μM for allopurinol, 0.15 to 20 μM for benznidazole, and 0.08 to 10 μM for nifurtimox. After 72 h, the cultures were fixed with methanol, stained with Giemsa, and examined microscopically to determine the percentage of cells infected in treated and untreated controls. IC_50_s and IC_90_s were calculated using Calcusyn software (Biosoft, United Kingdom). All experiments were run in duplicate, and the results were given as means ± standard deviations from at least two independent experiments.

### Allopurinol-nitroheterocyclic drug *in vivo* interaction.

Female Swiss mice (18 to 24 g) from the animal facility at the Federal University of Ouro Preto (UFOP), Minas Gerais, Brazil, were maintained in a temperature-controlled room with access to water and food *ad libitum* under a 12-h day/night cycle and at a temperature of 22 ± 2°C. The Ethics Committee in Animal Research at UFOP approved the procedures and experimental conditions (number 2009/17).

Animals inoculated with 5,000 blood trypomastigotes of the T. cruzi Y strain were randomly divided into 16 groups (*n* = 7 to 10/group). Animals were treated daily by oral gavage at doses of 25, 50, and 75 mg/kg of benznidazole and 30, 60, and 90 mg/kg of allopurinol in monotherapy for 20 consecutive days. Animals treated with the allopurinol and benznidazole combination received 30 mg of allopurinol plus 25, 50, and 75 mg/kg of benznidazole, 60 mg of allopurinol plus 25, 50, and 75 mg/kg of benznidazole, and 90 mg of allopurinol plus 25, 50, and 75 mg/kg of benznidazole. The 16th group of animals was treated with 100 mg/kg of benznidazole, the reference treatment for T. cruzi mouse infection. Finally, a group of infected animals receiving no treatment was used as a control. All treatments began at parasitemia onset, which occurred on day 4 postinfection.

### Treatment efficacy assessment.

Treatment efficacy was determined following the methodology of Caldas et al. (49) based on parasitemia detection by fresh blood examination before and after cyclophosphamide (Baxter Oncology, Germany) immunosuppression and blood qPCR. For qPCR, blood samples were collected 30 and 180 days after treatment from mice with negative fresh blood examination results. Animals showing negative results in all tests were considered cured.

### (i) Fresh blood examination and mortality.

The number of parasites in 5 μl of blood collected from the mouse tail vein was estimated (50). Mortality was checked daily until 30 days after treatment. Animals with negative results for fresh blood examination up to 30 days after treatment were immunosuppressed with cyclophosphamide at 50 mg/kg in 3 cycles of 4 consecutive days with an interval of 3 days between each cycle. Mice were checked daily for parasitemia during immunosuppression and for up to 10 days afterwards.

### (ii) Real-time PCR assay.

Isolation and purification of genomic DNA from blood samples were conducted using the Wizard genomic DNA purification kit (Promega Corp., Madison, WI) according to the manufacturer’s instructions. The presence of T. cruzi in blood samples was evaluated by amplifying a 195-bp tandem repeat in genomic DNA, using the following primers: TCZ-F (5′-GCTCTTGCCCACAMGGGTGC-3′, where M indicates A or C) and TCZ-R (5′-CCAAGCAGCGGATAGTTCAGG-3′), as described by Cummings and Tarleton (51). The murine tumor necrosis factor alpha (TNF-α) gene sequence was amplified separately using the primers TNF-5241 (5′-TCCCTCTCATCAGTTCTATGGCCCA-3′) and TNF-5411 (5′-CAGCAAGCATCTATGCACTTAGACCCC-3′) (51). Reaction mixtures consisted of 2 μl of template DNA, specific primers at a final concentration of 10 μM, and Sybr green PCR master mix in a total volume of 10 μl. DNA amplifications were carried out in an ABI 7300 real-time PCR system (Applied Biosystems, Life Technologies). After the initial denaturation step of 10 min at 95°C, amplification was carried out for 40 cycles (94°C for 15 s). Fluorescence data collection was performed at 64.3°C for 1 min at the end of each cycle. Amplification was immediately followed by a melting program with initial denaturation for 15 s at 95°C, cooling to 60°C for 1 min, and then a stepwise temperature increase from 60 to 95°C at 0.3°C/s. All samples were analyzed in duplicate, and negative samples and reagent controls were processed in parallel in each assay.

### (iii) IgG antibody detection.

T. cruzi-specific antibodies were detected in serum samples of mice collected 180 days after treatment, as described by Bahia et al. (52). In brief, enzyme-linked immunosorbent assay (ELISA) plates were coated with T. cruzi antigen prepared by alkaline extraction from the Y strain during exponential growth in liver infusion tryptose (LIT) medium. The sera were tested using both antigens and peroxidase-conjugated rabbit anti-mouse IgG (Bethyl Laboratory, USA). The mean values for absorbance at 490 nm for 10 negative-control samples were used to determine the reactivity index value, which was obtained by dividing the absorbance for each serum sample by the mean value for the control sample.

### Statistical analysis.

The nature of the interactions between allopurinol and nitroheterocyclic drugs *in vitro* was determined by fractional inhibitory concentration (FIC) index and isobologram construction. FICs at IC_50_ and the sum of FICs (ΣFICs) were calculated as follows: FIC of drug A = IC_50_ of drug A in combination/IC_50_ of drug A alone. The same equation was applied to the partner drug (drug B). ΣFICs = FIC drug A + FIC drug B. An overall mean ΣFIC was calculated for each combination and used to classify the nature of the interaction. Isobolograms were constructed, plotting the IC_50_ of allopurinol against those of benznidazole or nifurtimox. The ΣFIC_50_ was used to classify the interaction as synergism (ΣFIC ≤ 0.5), additive or no interaction (0.5 ≥ ΣFIC < 4), or antagonism (ΣFIC > 4) (53).

IgG antibody levels were analyzed by Mann-Whitney test performed by GraphPad Prism software. *P* values of *<*0.05 were considered to be significant.
